# "The Surgeon's Daughter," by Sir Walter Scott

**Published:** 1921-06-18

**Authors:** 


### HOSPITALS IN LITERATURE.

### II.—"The Surgeon's Daughter," by Sir Walter Scott.

It would be futile and absurd to attempt a comparison between the two novels "Nicholas Nickleby" and "The Surgeon's Daughter," or a comparison of any kind between Dickens and Scott. One star differeth from another star in glory. And yet anyone who has not read this short novel will, on reading the account of a "Species of Military Hospital," be irresistibly reminded of Dotheboys Hall. In both cases the institution is presided over by a savage one-eyed bully. Squeers enters the school preceded by Mrs. Squeers bearing two canes: Seelencooper marches round the main ward of his hospital armed with not only a "rattan," but also pistols and cutlass, and accompanied by men carrying handcuffs and strait jackets. At School and Hospital alike water is a rarity, and the diet is, to say the least, peculiar. Squeers buys, for the juvenile wretches committed to his care, the carcases of animals who have met with no violent death, while the state of things at the hospital may be gathered from an utterance of the superintendent, "I tell you the meat is as sweet as a nosegay." Again, the boys sleep five in a bed, and though at the hospital there are neither more nor less than two men as bed-fellows in each bed, yet at a certain spot only one of these two is alive.

### A Lovable Character.

It may be said, in passing, that the chapter containing the account of the hospital can be read and enjoyed, quite apart from the rest of the book, as a graphic and powerful piece of writing. "The Surgeon's Daughter" is far below Scott at his best. The country doctor (for "surgeon" has its old sense of general practitioner) is one of the most lovable characters in all the Waverley Novels, but the heroine is a mere name, serving only as a necessary wheel in the plot, or pawn in the game, and as a title for the book. These are by no means the only points for adverse criticism, yet the hospital chapter is in Scott's most lively descriptive and narrative style. There is always "atmosphere" in the great novelist's stories, but in his account of the "hospital" there is "atmosphere" in an intensified and even odoriferous sense.

The last adjective is created by the presence of sulphurous language, bad meat, bad gin, etc., *plus* one corpse, together with the novelist's own epithets—"den of horrors," "house of pestilence," etc.

An interesting literary question presents itself. Was Dickens influenced by this chapter of Scott in the picture of Dotheboys Hall? Did one suggest the other? It is well known that Dickens was a lover of the Waverley Novels, and we have not to travel outside "Nicholas Nickleby" itself to find a character, John Browdie, the Yorkshireman, who is strongly reminiscent of Dandie Dinmont (also Yorkshireman) in "Guy Mannering." But students of hospital history will perhaps be more concerned with such questions as the following: Did a hospital of this character, placed in the story at Ryde, Isle of Wight, exist there, or anywhere else, at the time (A.D. 1780)? Under what denomination of hospitals should it be classed? Does the account throw any light on the conditions of hospital life in general at that period?

Now it may be asserted with confidence that no such hospital ever existed at Ryde, Isle of Wight. This is the first reply, by the way, to the questioner who asked so indignantly in "Notes and Queries," October 4, 1873, "What was Scott's authority for the shocking description of the military hospital at Ryde?"

### The Ryde Hospital.

A reliable authority, residing in the Isle of Wight, writes to the present contributor, "No 'species of military hospital' ever existed at the time mentioned, even temporary in character. No earlier hospital at Ryde prior to the present county one." It is true that at the period in question a kind of hospital existed at many seaport towns, with special departments for sailors and soldiers on the move; but this is ruled out in the case of Ryde, (or Ride, and formerly La Rye), the place being then little more than a small fishing village, not at all approaching the rank of a seaport town. We hear of it in the seventeen-nineties ("Picture of the Isle of Wight Delineated upon the Spot," 1793, by H. P. W.) growing in popularity as a seaside resort and having "two tolerable inns." With regard to the question of classification, the index to "The Surgeon's Daughter" calls the alleged hospital a lazaretto, but if that is so Scott's own term of "a species of military hospital" must be abandoned. Perhaps "lazaretto" is a better selection from hospital nomenclature, for our purpose, though even that term is complimentary. The lines quoted from Milton at the head of the chapter should be noted:

> A lazar-house it seemed, wherein were laid
> Numbers of all diseased.

At the very date of the story the philanthropist, John Howard, well known for his exploration of prison abuses, was concerning himself actively with hospitals as well, but there is very little indeed in his reports on these institutions at all comparable with the abuses and horrors he found in prison life. In general, he seems to have been satisfied that hospitals on the whole were free from serious abuses, and the patients humanely treated, while Gray ("History of English Philanthropy") gives the same favourable verdict. One is driven to the conclusion that chapter vi. of "The Surgeon's Daughter," though written by an author with a strong feeling for historical background, is only fiction, and fiction of a highly imaginative kind at that. Perhaps, however, it may be true to say that it was designed, apart from its natural sequence in the story, to illustrate abuses in the military system of that time, especially in the matter of transport. It is also true that Scott succeeds in making it appear that his bogus hospital is based on historical fact. Such verisimilitude is one of the highest forms of art.

